# Supplementary material for: mRNA vaccines and hybrid immunity use different B cell germlines against Omicron BA.4 and BA.5
Source: Nat Commun. 2023 Mar 28;14:1734. doi: 10.1038/s41467-023-37422-y (PMC10044118; doi:10.1038/s41467-023-37422-y)
Supplement: Supplementary file 1 — Supplementary Information [file 41467_2023_37422_MOESM1_ESM.pdf]

SUPPLEMENTARY INFORMATION

mRNA vaccines and hybrid immunity use different B cell germlines against Omicron BA.4 and BA.5

Emanuele Andreano[1], Ida Paciello[1], Giulio Pierleoni[2], Giuseppe Maccari[3], Giada Antonelli[1], Valentina Abbiento[1], Piero Pileri[1], Linda Benincasa[2], Ginevra Giglioli[2], Giulia Piccini[4], Concetta De Santi[1], Claudia Sala[1], Duccio Medini[3], Emanuele Montomoli[2,4,5], Piet Maes[6], Rino Rappuoli[7,8,*]

[1]Monoclonal Antibody Discovery (MAD) Lab, Fondazione Toscana Life Sciences, Siena, Italy

[2]VisMederi Research S.r.l., Siena, Italy

[3]Data Science for Health (DaScH) Lab, Fondazione Toscana Life Sciences, Siena, Italy

[4]VisMederi S.r.l, Siena, Italy

[5]Department of Molecular and Developmental Medicine, University of Siena, Siena, Italy

[6]KU Leuven, Rega Institute, Department of Microbiology, Immunology and Transplantation, Laboratory of Clinical and Epidemiological Virology, Leuven, Belgium

[7]Department of Biotechnology, Chemistry and Pharmacy, University of Siena, Siena, Italy

[8]Fondazione Biotecnopolo di Siena, Siena, Italy

*Corresponding author: Rino Rappuoli rino.rappuoli@biotecnopolo.it

SUPPLEMENTARY FIGURES

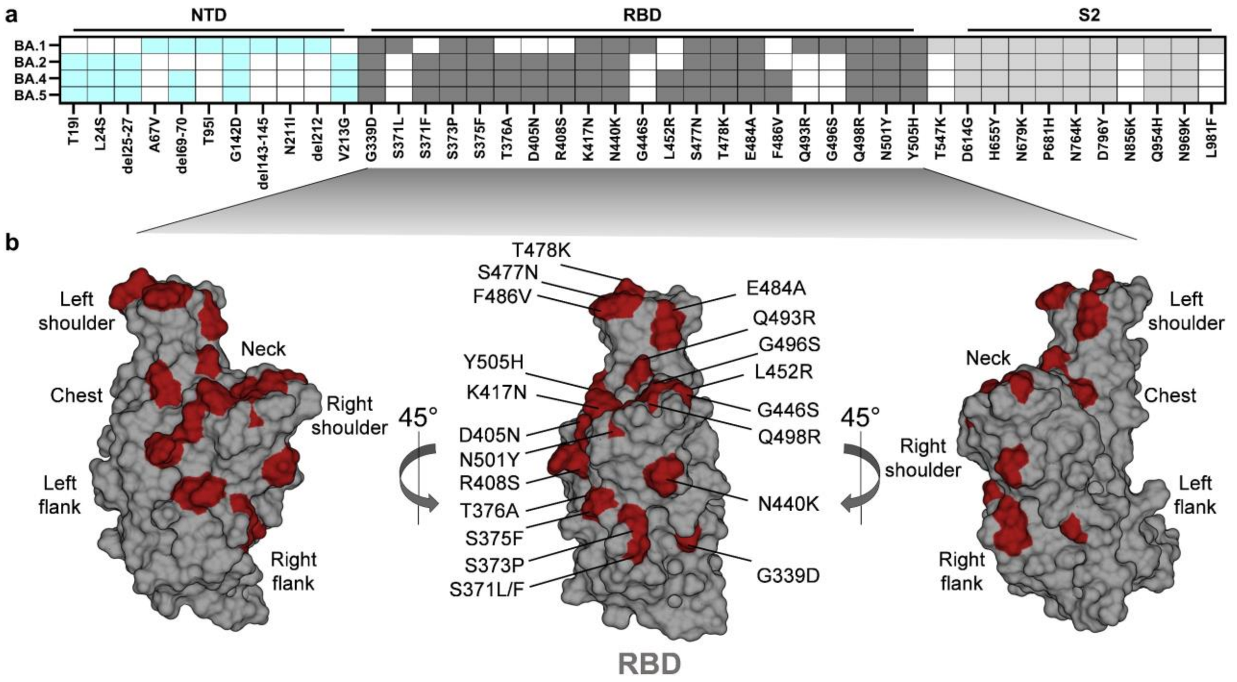

Supplementary Fig. 1. Distribution of omicron mutations on SARS-CoV-2 S protein. a, the heatmap shows the SARS-CoV-2 S protein mutations in the NTD (cyan), RBD (dark gray) and S2 domain (light gray) of omicron BA.1, BA.2, BA.4 and BA.5. **b**, Representation of the RBD with all omicron mutations highlighted in red.

a

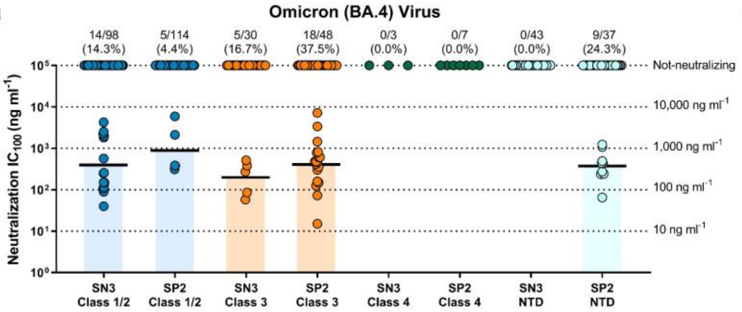

c

Cohort	Class 1/2	Class 3	Class 4	NTD
Seronegative 3rd dose (SN3) GM-IC_{100} ng ml^{-1}	402.6	192.1	N/A	N/A
Seropositive 2nd dose (SP2) GM-IC_{100} ng ml^{-1}	891.7	413.1	N/A	367.9

b

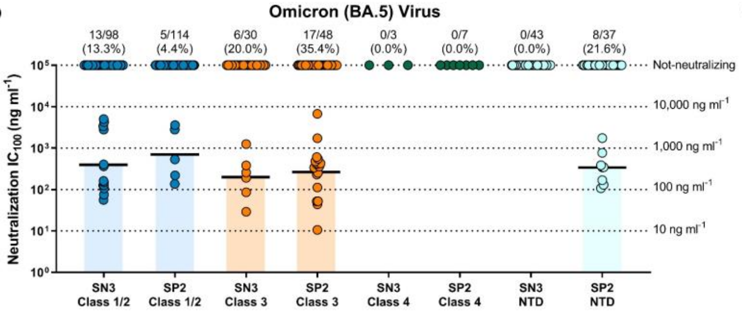

d

Cohort	Class 1/2	Class 3	Class 4	NTD
Seronegative 3rd dose (SN3) GM-IC_{100} ng ml^{-1}	390.3	196.5	N/A	N/A
Seropositive 2nd dose (SP2) GM-IC_{100} ng ml^{-1}	692.4	262.5	N/A	330.3

Supplementary Fig. 2. Distribution and neutralization potency of RBD and NTD-targeting nAbs against omicron BA.4 and BA.5. a-b, dot charts compare the distribution of nAbs isolated from subjects belonging to the SN3 and SP2 cohorts against omicron BA.4 (**a**) and BA.5 (**b**) omicron variants. The number, percentage and GM-IC_{100} (black lines and colored bars) of nAbs are denoted on each graph. **c-d**, table summarizing the GM-IC_{100} of Class 1/2, Class 3, Class 4 and NTD nAbs against BA.4 (**c**) and BA.5 (**d**). Source data are provided as a Source Data file.

SUPPLEMENTARY TABLES

Supplementary Table 1. Numbers and frequency of nAbs in the SN3 cohort.

Subject	Vaccine	Wuhan – Neutralizing antibodies	Omicron BA.1 – Neutralizing antibodies (%)	Omicron BA.2 – Neutralizing antibodies (%)	Omicron BA.4 – Neutralizing antibodies (%)	Omicron BA.5 – Neutralizing antibodies (%)
VAC-001	BNT162b2	106	22 (20.8)	24 (22.6)	11 (10.4)	9 (8.5)
VAC-002	BNT162b2	57	15 (26.3)	17 (29.8)	9 (15.8)	9 (15.8)
VAC-008	BNT162b2	36	7 (19.4)	9 (25.0)	4 (11.1)	5 (13.9)
VAC-010	mRNA-1273	7	1 (14.3)	2 (28.6)	0 (0.0)	0 (0.0)
	**Total**	**206**	**45 (21.8)**	**52 (25.2)**	**24 (11.6)**	**23 (11.2)**

Supplementary Table 2. Frequency of predominant germlines against omicron variants.

Seronegative 2nd doses (SN2)

Germlines	Wuhan Virus (%)	Omicron (BA.1) (%)	Omicron (BA.2) (%)	Omicron (BA.4) (%)	Omicron (BA.5) (%)
IGHV1-69;IGHJ4-1	2 (4.3)	0 (0.0)	0 (0.0)	0 (0.0)	0 (0.0)
IGHV3-30;IGHJ6-1	2 (4.3)	0 (0.0)	0 (0.0)	0 (0.0)	0 (0.0)
IGHV3-53;IGHJ6-1	6 (13.0)	0 (0.0)	1 (25.0)	0 (0.0)	0 (0.0)
IGHV3-66;IGHJ4-1	3 (6.5)	0 (0.0)	0 (0.0)	0 (0.0)	0 (0.0)
Tot. nAbs	46	1	4	0	0
Tot. germline specific nAbs	13 (28.3)	0 (0.0)	1 (25.0)	0 (0.0)	0 (0.0)

Seronegative 3rd doses (SN3)

Germlines	Wuhan Virus (%)	Omicron (BA.1) (%)	Omicron (BA.2) (%)	Omicron (BA.4) (%)	Omicron (BA.5) (%)
IGHV1-58;IGHJ3-1	12 (6.8)	5 (13.9)	4 (9.5)	1 (4.5)	0 (0.0)
IGHV1-69;IGHJ3-1	10 (5.7)	4 (11.1)	5 (11.9)	1 (4.5)	3 (13.6)
IGHV1-69;IGHJ4-1	16 (9.1)	9 (25.0)	9 (21.4)	4 (18.2)	4 (18.2)
IGHV3-66;IGHJ4-1	9 (5.1)	0 (0.0)	1 (2.4)	2 (9.1)	3 (13.6)
IGHV3-66;IGHJ6-1	10 (5.7)	3 (8.3)	4 (9.5)	4 (18.2)	3 (13.6)
Tot. nAbs	176	36	42	22	22
Tot. germline specific nAbs	57 (32.4)	21 (58.3)	23 (54.8)	12 (54.6)	13 (59.1)

Seropositive 2nd doses (SP2)

Germlines	Wuhan Virus (%)	Omicron (BA.1) (%)	Omicron (BA.2) (%)	Omicron (BA.4) (%)	Omicron (BA.5) (%)
IGHV1-24;IGHJ6-1	5 (2.4)	0 (0.0)	3 (6.1)	3 (10.0)	3 (10.3)
IGHV1-58;IGHJ3-1	10 (4.8)	7 (18.9)	5 (10.2)	0 (0.0)	0 (0.0)
IGHV2-5;IGHJ4-1	9 (4.3)	2 (5.4)	6 (12.2)	6 (20.0)	6 (20.7)
Tot. nAbs	208	37	49	30	29
Tot. germline specific nAbs	24 (11.5)	9 (24.3)	14 (28.6)	9 (30.0)	9 (31.0)

Supplementary Table 3. Neutralization potency of predominant germlines against omicron variants.

Germlines	Wuhan Virus GM-IC_{100} ng ml^{-1}	Omicron (BA.1) GM-IC_{100} ng ml^{-1}	Omicron (BA.2) GM-IC_{100} ng ml^{-1}	Omicron (BA.4) GM-IC_{100} ng ml^{-1}	Omicron (BA.5) GM-IC_{100} ng ml^{-1}
**Seronegative 2nd doses (SN2)**					
IGHV1-69;IGHJ4-1	191.63	N/A	N/A	N/A	N/A
IGHV3-30;IGHJ6-1	382.71	N/A	N/A	N/A	N/A
IGHV3-53;IGHJ6-1	79.58	N/A	25.60	N/A	N/A
IGHV3-66;IGHJ4-1	66.42	N/A	N/A	N/A	N/A
**Seronegative 3rd doses (SN3)**					
IGHV1-58;IGHJ3-1	93.79	157.36	192.01	4261.36	N/A
IGHV1-69;IGHJ3-1	50.21	155.79	124.13	39.67	616.76
IGHV1-69;IGHJ4-1	212.38	987.84	1069.88	1318.53	1927.94
IGHV3-66;IGHJ4-1	327.61	N/A	66.17	253.58	578.35
IGHV3-66;IGHJ6-1	272.30	295.43	223.63	494.97	453.70
**Seropositive 2nd doses (SP2)**					
IGHV1-24;IGHJ6-1	314.04	N/A	130.20	413.36	463.99
IGHV1-58;IGHJ3-1	52.19	852.49	982.36	N/A	N/A
IGHV2-5;IGHJ4-1	28.10	379.68	197.85	264.09	166.37
