# Supplementary material for: mRNA vaccines and hybrid immunity use different B cell germlines against Omicron BA.4 and BA.5
Source: Nat Commun. 2023 Mar 28;14:1734. doi: 10.1038/s41467-023-37422-y (PMC10044118; doi:10.1038/s41467-023-37422-y)
Supplement: Supplementary file 3 — Reporting Summary [file 41467_2023_37422_MOESM3_ESM.pdf]

nature portfolio

Corresponding author(s): Rino Rappuoli

Last updated by author(s): Mar 1, 2023

# Reporting Summary

Nature Portfolio wishes to improve the reproducibility of the work that we publish. This form provides structure for consistency and transparency in reporting. For further information on Nature Portfolio policies, see our Editorial Policies and the Editorial Policy Checklist.

## Statistics

For all statistical analyses, confirm that the following items are present in the figure legend, table legend, main text, or Methods section.

| n/a | Confirmed | |
|---|---|---|
| ☐ | ☒ | The exact sample size (*n*) for each experimental group/condition, given as a discrete number and unit of measurement |
| ☐ | ☒ | A statement on whether measurements were taken from distinct samples or whether the same sample was measured repeatedly |
| ☐ | ☒ | The statistical test(s) used AND whether they are one- or two-sided<br>*Only common tests should be described solely by name; describe more complex techniques in the Methods section.* |
| ☒ | ☐ | A description of all covariates tested |
| ☒ | ☐ | A description of any assumptions or corrections, such as tests of normality and adjustment for multiple comparisons |
| ☐ | ☒ | A full description of the statistical parameters including central tendency (e.g. means) or other basic estimates (e.g. regression coefficient) AND variation (e.g. standard deviation) or associated estimates of uncertainty (e.g. confidence intervals) |
| ☐ | ☒ | For null hypothesis testing, the test statistic (e.g. *F*, *t*, *r*) with confidence intervals, effect sizes, degrees of freedom and *P* value noted<br>*Give P values as exact values whenever suitable.* |
| ☒ | ☐ | For Bayesian analysis, information on the choice of priors and Markov chain Monte Carlo settings |
| ☒ | ☐ | For hierarchical and complex designs, identification of the appropriate level for tests and full reporting of outcomes |
| ☒ | ☐ | Estimates of effect sizes (e.g. Cohen's *d*, Pearson's *r*), indicating how they were calculated |

*Our web collection on statistics for biologists contains articles on many of the points above.*

## Software and code

Policy information about availability of computer code

| | |
|---|---|
| Data collection | No software was used for data collection. |
| Data analysis | - GraphPad Prism 8.0.2 was used to perform statistical analyses<br>- Boston University, Cloanalyst (http://www.bu.edu/computationalimmunology/research/software/) 0.9.0.0<br>- ggplot2 v3.3.5<br>- CLC sequence viewer v8.1 |

For manuscripts utilizing custom algorithms or software that are central to the research but not yet described in published literature, software must be made available to editors and reviewers. We strongly encourage code deposition in a community repository (e.g. GitHub). See the Nature Portfolio guidelines for submitting code & software for further information.

## Data

Policy information about availability of data

All manuscripts must include a data availability statement. This statement should provide the following information, where applicable:

- Accession codes, unique identifiers, or web links for publicly available datasets
- A description of any restrictions on data availability
- For clinical datasets or third party data, please ensure that the statement adheres to our policy

Source data are provided with this paper. All data supporting the findings in this study are available within the article or can be obtained from the corresponding

author upon request. SARS-CoV-2 antibody sequences were deposited and accessible from https://github.com/dasch-lab/SARS-CoV-2_nAb_third_dose.

## Human research participants

Policy information about studies involving human research participants and Sex and Gender in Research.

| | |
|---|---|
| Reporting on sex and gender | In this study we evaluated three different cohorts. Five subjects were enrolled in both the seronegative 2nd dose (SN2; n = 5) and seropositive 2nd dose (SP2; n = 5) cohorts. Four subjects were enrolled in the seronegative 3rd dose cohort (SN3; n = 4). The female to male ratio for each cohort was 2:3, 2:2 and 2:3 for SN2, SN3 and SP2 respectively. |
| Population characteristics | The age of donors enrolled in the three cohorts ranged from 25 to 57 years (GM of 41.3, 43.2 and 36.2 years of age for SN2, SN3 and SP2 respectively)16,17. No statistical methods were used to predetermine sample size. |
| Recruitment | Partecipants were selected based on their immunological history, therefore two vaccine doses for SN2, three vaccine foses for (SN3) and infection prior to vaccination for SP2. |
| Ethics oversight | Human samples from SARS-CoV-2 infected and vaccinated donors, who received two or three vaccine doses, of both sexes, were previously collected through a collaboration with the Azienda Ospedaliera Universitaria Senese, Siena (IT) (DOI: 10.1038/s41586-021-04117-7; DOI: 10.1038/s41467-022-35781-6). All subjects enrolled gave their written consent. The study that allowed the enrollment of subjects in all three cohorts was approved by the Comitato Etico di Area Vasta Sud Est (CEAVSE) ethics committees (Parere 17065 in Siena) and conducted according to good clinical practice in accordance with the declaration of Helsinki (European Council 2001, US Code of Federal Regulations, ICH 1997). |

Note that full information on the approval of the study protocol must also be provided in the manuscript.

# Field-specific reporting

Please select the one below that is the best fit for your research. If you are not sure, read the appropriate sections before making your selection.

☒ Life sciences ☐ Behavioural & social sciences ☐ Ecological, evolutionary & environmental sciences

For a reference copy of the document with all sections, see nature.com/documents/nr-reporting-summary-flat.pdf

# Life sciences study design

All studies must disclose on these points even when the disclosure is negative.

| | |
|---|---|
| Sample size | We analyzed of 482 human neutralizing antibodies isolated from 14 previously enrolled COVID-19 vaccinated subjects (5 seronegative 2nd dose, 4 seronegative 3rd dose and 5 seropositive), to evaluate cross-protection of vaccine induced-antibodies against SARS-CoV-2 omicron BA.1, BA.2, BA.4 and BA.5. Given the exploratory nature of the study, we did not use statistical methods to predetermine sample size of human monoclonal antibodies tested per each group. Sample size was based on previous studies that applied a similar technology (DOI: 10.1038/s41586-021-04117-7; DOI: 10.1038/s41467-022-35781-6). 4-5 subjects/group were selected as they represent a good balance between feasibility of analyzing at single cell level several thousands of memory B cells and the ability to represent the antibody response of seronegative and seropositive people. |
| Data exclusions | No data was excluded. |
| Replication | All experiments were performed in technical duplicates or triplicates as indicated in the figure legends and methods section. |
| Randomization | The experiments were not randomized and all available samples were tested. The authors aimed to specifically assess the antibody response of seronegative and seropositve subjects. Based on what stated above, the authors believed that randomization was not appropriate. |
| Blinding | The aim of our study was to evaluate the antibody response of three specific cohorts, subjects vaccinated with two or three vaccine doses, or vaccinated after infection. Therefore, to be sure that donors were properly allocated to the correct cohort, the investigators were not blinded during group allocation, data collection and analyses. |

# Reporting for specific materials, systems and methods

We require information from authors about some types of materials, experimental systems and methods used in many studies. Here, indicate whether each material, system or method listed is relevant to your study. If you are not sure if a list item applies to your research, read the appropriate section before selecting a response.

## Materials & experimental systems

| n/a | Involved in the study |
|---|---|
| [x] | [ ] Antibodies |
| [ ] | [x] Eukaryotic cell lines |
| [x] | [ ] Palaeontology and archaeology |
| [x] | [ ] Animals and other organisms |
| [x] | [ ] Clinical data |
| [x] | [ ] Dual use research of concern |

## Methods

| n/a | Involved in the study |
|---|---|
| [x] | [ ] ChIP-seq |
| [x] | [ ] Flow cytometry |
| [x] | [ ] MRI-based neuroimaging |

# Eukaryotic cell lines

Policy information about cell lines and Sex and Gender in Research

| | |
|---|---|
| Cell line source(s) | VERO E6 cell line ATCC Cat#CRL-1586; Expi293F cells Thermo Fisher Cat#A14527 |
| Authentication | These cell lines were obtained from vendors that sell authenticated cell lines, they grew, performed and showed morphology as expected. No additional specific authentication was performed. |
| Mycoplasma contamination | Vero E6 cell lines are routinely tested on a monthly basis and tested negative for mycoplasma. Expi293F cells were not tested for mycoplasma contamination. |
| Commonly misidentified lines (See ICLAC register) | No commonly misidentified cell lines were used in this study. |
